# Psychometric Properties of the Slovak Version of Short Dark Triad

**DOI:** 10.3390/ejihpe11030047

**Published:** 2021-07-03

**Authors:** Radka Čopková, Leoš Šafár

**Affiliations:** 1 Departament of Economics, Faculty of Economics, Technical University of Košice, 040 01 Košice, Slovakia; 2 Department of Banking and Investment, Faculty of Economics, Technical University of Košice, 040 01 Košice, Slovakia; leos.safar@tuke.sk

**Keywords:** Dark Triad, Machiavellianism, narcissism, psychopathy, Short Dark Triad, psychometric properties

## Abstract

The Short Dark Triad is a scale used to capture three aversive personality traits—Machiavellianism, narcissism, and psychopathy on the subclinical level. The present study aimed to verify the psychometric properties of the Slovak version of the Short Dark Triad scale in three studies. The first two studies aimed to examine the reliability of the scale. The aim of Study 1 was to examine the factor structure of SD3. A three-factor model consisting of three latent intercorrelated factors in a unidimensional and bifactorial model were examined on a sample of 588 participants. Study 2 aimed to test the consistency of the results over time (test–retest reliability) on the sample of 117 participants. In Study 3, convergent and divergent validity was examined on the sample of 333 participants. For both kinds of validity examination, the Slovak version of NEO-FFI was used. The internal consistency of the subscales and test results, the same as the retest results, were satisfactory. The relationships between the scales were found to be significant. Confirmatory factor analysis (CFA) results supported the original three-factor model. Significant interrelations have been established between Machiavellianism and openness, agreeableness and conscientiousness; narcissism and neuroticism, extraversion and agreeableness; psychopathy and openness, agreeableness and conscientiousness. The Short Dark Triad achieved satisfactory values of reliability and validity; therefore, it can be used on the Slovak population.

## 1. Introduction

The interest of humankind in knowing and understanding the human personality has a long history. In the past, the focus was on creating the concept of personality in its horizontal (theoretical) structuring (e.g., Hippocrates, Pavlov, Jung or Constitutional typology) and vertical (analytical) structuring (e.g., the theories of S. Freud, E. Erickson), whereas nowadays, there is more emphasis on the objective (psychometric) identification of essential personality traits, which has created an opportunity for the emergence of a factor approach to personality structuring (e.g., Eysenck’s model, Cattel’s model, Big Five). The factor structure of personality explains the existence of personality traits in a dimensional context, which, so far, is the best way to reflect the idea that each normal individual has a whole spectrum of personality traits, while each of those traits moves in its dimension on a continuum between two opposites, whereby each individual becomes the bearer of his own unique configuration of these traits [1,2].

Focusing more on the factor concepts of personality, it is possible to notice an emphasis on adaptive personality traits in their interpretation—the so-called “brighter” side of a personality. Such a concept has been described as the Big Five model by Oluf and Furnham [3]. However, less attention has been paid to the fact that each person has, to some extent, maladaptive personality traits—the so-called "dark" side of personality. The concept that has attracted experts’ attention in recent years is the concept of The Dark Triad [4]. The obvious issue that arose after Dark Triad conceptualization was to develop a tool which would be able to capture it and enable measurements of the extent of aversive traits in human personality structure. In 2014, Jones and Paulhus [1] developed a brief scale—the Short Dark Triad. Consequently, other language versions have been developed, so the brief scale could be used in different cultures. However, in Slovakia, the formal tool is still missing, which led authors of this study to verify the psychometric properties of the Slovak version of the Short Dark Triad scale.

## 2. Dark Triad

Related Dark Triad research has an approximately 20-year history, which brought new insights to the subject of the dark sides of personality. Traits in the Dark Triad concept share certain common characteristics [4]. They overlap in attributes such as self-assertion, emotional callousness, aggression, lack of sincerity, and absence of humility [4,5,6]. At this point, it should be emphasized that these are subclinical traits—the behavior of individuals who exhibit these characteristics is not extreme enough to attract the attention of clinical psychologists or psychiatrists. Thanks to the excellent ability of bearers of dark traits to adapt and the lower intensity of these negative personality traits, these individuals are a common part of the wider society and everyday life.

Narcissism is understood as a stable personality trait [7]. Subclinical narcissists show signs of exaggerated self-love, attention and admiration requirement, inflated self-confidence, a sense of importance and superiority over others, and lack of interest in others. Morf and Rhodewalt [8] claim that narcissists have an extremely positive, but at the same time, a vulnerable self-image. Outwardly, they aim to display their invincibility in front of others.

Machiavellianism describes a personality that is characterized by emotional separation and a tendency to manipulate, in order to achieve one’s own goal regardless of others [9]. Machiavellians are systematic, they build alliances, and do everything they can to maintain a positive reputation [1]. They disseminate false information about themselves, aiming to create a false illusion of intimacy. They are very good liars, but they know how to manipulate people so cleverly that they cannot ultimately be considered completely bad, because they do not break the rules completely, but have an exceptional ability to circumvent them skillfully [10].

Subclinical psychopathy has been identified in the Dark Triad as the most destructive [11]. It is characterized by features such as high impulsivity, excitement seeking, low empathy, low degree of anxiety [4], lack of concern for others, lack of guilt when they hurt others, and emotional shallowness [12]. Other people are most often perceived as rivals, enemies, or threats [6].

Within the studies, Dark Triad traits are often compared to Big Five traits—neuroticism, extraversion, openness, agreeableness, conscientiousness. In the following lines, we provide a brief overview of previous studies that have addressed the relationship between the concepts of the Dark Triad and the Big Five.

Jakobwitz and Egan [13] and Furnham et al. [14] positively associated Machiavellianism with neuroticism and negatively associated it with conscientiousness and agreeableness; they positively associated narcissism with extraversion, openness and conscientiousness and negatively associated it with agreeableness; and they positively associated psychopathy with neuroticism and negatively associated it with conscientiousness and agreeableness. 

The Big Five features are very often used as a validation measure for scales capturing Dark Triad traits. In this context, Malesza et al. [15] found negative relationships of Machiavellianism and psychopathy with conscientiousness and agreeableness, whereas narcissism correlated positively with conscientiousness and negatively with agreeableness. In a study by Mejzlíková et al. [16], a negative relationship between Machiavellianism and agreeableness was found. Narcissism had a negative relationship with neuroticism and agreeableness, and on the other hand, a positive relationship with extraversion and openness. Psychopathy correlated positively with neuroticism and negatively with agreeableness and conscientiousness. In the study by Odiakosa [17], Machiavellianism had a negative relationship with agreeableness; narcissism correlated negatively with neuroticism and positively with extraversion; and psychopathy correlated positively with neuroticism and negatively with agreeableness. The authors of the concept of Dark Triad, Paulhus and Williams [4], point out the negative relationships of Machiavellianism with agreeableness and conscientiousness. They associated narcissism positively with extraversion and openness, and negatively with agreeableness. Finally, they interpreted the positive relationships of psychopathy with extraversion and openness, and the negative relationships with neuroticism, conscientiousness and agreeableness. Table 1 briefly summarizes the abovementioned conclusions.

## 3. Measures of Dark Triad

Given that the very concept of the Dark Triad emerged long after its components were known to the experts, it is not surprising that several questionnaires and various scales have been developed and used to capture them.

Undoubtedly, the most widely used methodology for capturing subclinical narcissism is the 40-item Narcissistic Personality Inventory (NPI) [18] and its abbreviated 16-item version (NPI-16) [19]. This scale captures the constant search for attention, extreme pettiness, excessive self-centeredness, and exploitation in interpersonal relationships [13].

In research focused on capturing the manifestations of Machiavellianism, a methodology called MACH [20] has a long tradition—currently, the sixth revision (MACH-VI) is being processed, although its fourth revision (MACH-IV) is the most widely applied. The main characteristics representing the Machiavellian orientation relate to interpersonal tactics, including the manipulation of others, insight into human nature and abstract morality. In order to eliminate certain shortcomings of this methodology, Dahling et al. [21] developed a methodology that captures Machiavellianism on a more personal level. They described the Machiavellian personality through four key characteristics—distrust of other people, amoral manipulation, desire for control, and desire for status.

The Psychopathy Check List [22] was used as a standard to measure psychopathy. Subsequently, a 64-item Psychopathy Self-Report scale [23] was developed. It was compiled from items that distinguished clinically diagnosed psychopaths from subclinical psychopaths. There are two main factors: the first relates to the interpersonal and affective aspects of psychopathy; the second to the social deviation associated with psychopathy [24].

Although the abovementioned questionnaires and scales continue to be popular and widely used, possible shortcomings have emerged from the number of items contained. With the emerging Dark Triad concept and a consequent effort to capture all containing components, briefer measurements have become a necessity.

Two methodologies are often adopted. The first is Dirty Dozen [25], consisting of 12 items grouped in 3 factors (each factor consists of 4 items); secondly, the 27-item Short Dark Triad (SD3) scale was created. Briefly, we present several studies that aimed to compare the Dirty Dozen [25] and Short Dark Triad [1].

Muris et al. [26] pointed out that both scales are rather scarce regarding the items contained; therefore, the ability to fully reflect the features of the Dark Triad is limited. In the case of Dirty Dozen, we found ambiguous results about its convergent validity. Some studies suggested that Dirty Dozen disposed of problematic convergent validity, for which it was criticized [15,27,28]. Other studies have recommended it as a useful tool [29]. Maples et al. [27] emphasized that the Short Dark Triad has better convergent validity [27] compared to Dirty Dozen; therefore, they recommended Short Dark Triad as more consistent tool.

Furnham et al. [14] reported the existence of two more attempts to capture the Dark Triad—the Dark Triad Screening Measure [30], which attempted to create subscales with minimal overlap, and Mini-Markers of Evil [31], formed exclusively by adjectives reflecting individual features of the Dark Triad.

### Short Dark Triad (SD3)

The abovementioned information suggests that there is great interest in developing a valid and reliable methodology for capturing the phenomenon of the Dark Triad. The last attempt was made by Jones and Paulhus [1], who created the Short Dark Triad in 2014.

Based on a theoretical background, the authors created a list of 41 items that were potentially saturated with three factors of the Dark Triad—narcissism, Machiavellianism, and psychopathy. The subscale of narcissism corresponded to 13 items that saturated characteristics of narcissistic personality such as leadership, exhibitionism, grandiosity, and claim. The subscale of Machiavellianism was also made up of 13 items, reflecting tendencies to form alliances, planning, manipulation, cynicism, and reputation. The psychopathy subscale consisted of 15 items that corresponded to antisocial behavior, unstable lifestyle, heartlessness, and short-term manipulation. The exploratory factor analysis selected 14 items that either did not sufficiently saturate any of the factors or saturated more than one, which meant that there was a significant overlap of constructs (three items from the subscale of Machiavellianism and one item from the subscale of narcissism, which saturated the subscale of psychopathy). A list of 27 items resulted from this analysis, where each of the subscales consisted of nine items. This factor structure was also confirmed by the analysis of the main components. The reliability of the subscales was proven to be sufficient (greater than 0.7). Subscale intercorrelations were evaluated as positive and moderate, but significant.

The factor structure of the Short Dark Triad was examined using exploratory modelling of structural equations on two samples. The model fit for the first sample was acceptable, but for the second sample, the model fit was slightly weaker. However, the authors stated that it was still acceptable because all items saturated their reference factors.

Discriminatory validity was examined on standard methodologies (MACH IV, NPI, SRP, Dirty Dozen) and considered as satisfactory.

The Short Dark Triad scale [1] has also attracted the attention of experts in other countries who tried to adapt it.

Other modifications of the Short Dark Triad have been created for Czech [16], German [15], Argentinian [32], Turkish [33], Russian [34], Serbian [35], Thai [36], Polish [37], Japanese [38], Chinese [39], Iranian [40] and Spanish [41] populations. The approaches of the abovementioned researchers differ in some aspects; for example, in the models of factor structure (unidimensional model, correlated two-factor model, correlated three-factor model, bifactor model) or number of items in the final version of the language-modified scale (for example, the Czech version consisted of 29 items, Argentinian of 2 items, Thai of 25 items, Iranian of 20 items, etc.).

Thus, it is clear that the scale is gradually being adapted to several languages. The aim of the present study was to verify the psychometric properties of the Slovak version of the Short Dark Triad, because according to available sources, an adapted Slovak version is still absent. This will expand the repertoire of language variations of this scale. Despite this, the scale is increasingly used in Slovak research projects. We attempted to achieve our goals in three studies.

## 4. Study 1—Factor Structure of the Slovak Short Dark Triad

The aim of the first study was to examine the factor structure of SD3. A three-factor model consisting of three latent intercorrelated factors—Machiavellianism, narcissism and psychopathy—was examined. This model corresponds to the model as proposed by the primary authors [1].

### 4.1. Method

#### 4.1.1. Participants

The first study involved 588 participants aged 17 to 65 years (M_Age_ = 27.6; SD = 11.5 years). The gender representation of the respondents was as follows—men made up 34.3% (N = 202) of the research group and were aged 17 to 62 years (M_Age_ = 24.6; SD = 9.76); women accounted for 65.7% (N = 386) of the study population and were aged 17 to 65 years (M_Age_ = 29.6; SD = 12.2). A convenience and purposive sampling method was used.

#### 4.1.2. Measures

The Short Dark Triad (SD3) is a self-assessment scale made up of 27 statements that reflect aversive personality traits. The respondents are expected to express their degree of agreement on a 5-point Likert scale (1 = strongly disagree; 5 = strongly agree) with the statements. Statements evenly saturated three factors: nine items saturated Machiavellianism (e.g., “*I like to use clever manipulation to get my way*”); nine items saturated narcissism (e.g., “*I know that I am special because everyone keeps telling me so*”); and nine items saturated psychopathy (e.g., “*Payback needs to be quick and nasty*”). Each of the subscales were evaluated separately, i.e., for each subscale, a total score was calculated by the sum of the points that the respondent marked in the individual answers. Due to the width of the scale, the respondent could score each of the subscales in the range of 9 to 45 points. The higher the number of points they achieved, the more they reflected the qualities corresponding to a particular aversive personality trait. Some of the items were formulated in reverse, they had to be reverse-coded before calculating the total score. These were three items from the subscale of narcissism, numbers 11, 15, and 17 (“*I hate being the center of attention.*”) and two items from the subscale of psychopathy, numbers 20 and 25 (“*I have never gotten into trouble with the law.*”).

The original version of the questionnaire is freely available in the profile of the original authors on the portal www.researchgate.com as well as on the Google Scholar domain and in the Web of Science database.

The Slovak version of the questionnaire, called the “Krátka škála temnej triády”, was created by translating the original Short Dark Triad Scale (SD3) published in English. The translation was performed by three experts. Consequently, the Slovak version was translated back to English by a language specialist, with the aim of eliminating linguistic ambiguities and optimize the most accurate version. The Slovak version of the questionnaire is given in the appendix to this study (Appendix A).

#### 4.1.3. Procedure and Statistical Analyses

The questionnaire was distributed to the respondents through the period September 2018–April 2019 in two different forms. The electronic version of the questionnaires was created using the Google Docs-Form web application and sent via e-mail. The second version was in printed form (‘pencil–paper’). Students and teachers from Slovak universities were contacted, and they were asked not only to fill in the questionnaire, but also to distribute it further. Respondents were informed that the completion of the questionnaire was voluntary and anonymous, and the data would only be processed in this research study. All respondents agreed to participate in the research.

The collected data were subjected to statistical analysis in IBM SPSS Statistics 21, Jamovi 0.8.1.13 and R 3.6.2.

There were no missing data from the data file. Testing the normality of data distribution using the Kolmogorov–Smirnov test showed that the data were not normally distributed (*p* < 0.05). The values of skewness and kurtosis did not exceed the criterion of >±1. The internal consistency of the Short Dark Triad subscales was determined using McDonald’s omega. Descriptive statistics were used (mean, median, standard deviation, minimum, maximum). We evaluated the intercorrelations of the subscales of the Short Dark Triad using the Spearman correlation coefficient. Gender differences were tested using a t-test for independent samples. In the confirmatory factor analysis, we evaluated the following model fit indicators—chi-squared (χ^2^), Tucker–Lewis index (TLI), comparative fit index (CFI), and the root-mean-square error of approximation (RMSEA).

### 4.2. Results

Table 2 shows the values of the basic descriptive indicators for all three subscales of the Short Dark Triad—Machiavellianism, narcissism, and psychopathy.

The reliability of individual subscales was calculated using McDonald’s omega. The subscale of Machiavellianism had a value of ω_m_ = 0.733; the subscale of narcissism had a value of ω_n_ = 0.667 and the subscale of psychopathy had a value of ω_p_ = 0.728. The item analysis (Table 3) suggests that discarding M1, N9, and P2 could improve the values for the internal consistency of each of the subscales. Such removal of the aforementioned items did not result in a substantial increase in values; therefore, we decided to use the whole set of items for further analysis, i.e., 27.

The theoretical basis of the original model of the Dark Triad suggests that the individual dimensions overlap in some features, suggesting that there should be significant relationships between them. Spearman’s correlation coefficient confirmed this assumption; it reached ρ_m−n_ = 0.369 (*p* < 0.001; 95% CI [0.297; 0.437]); ρ_m−p_ = 0.596 (*p* <0.001; 95% CI [0.541; 0.645]); and ρ_n−p_ = 0.400 (*p* < 0.001; 95% CI [0.329; 0.466]).

Subsequently, gender difference testing was conducted, because it is usually a part of Short Dark Triad adaptation. The results showed that male respondents scored significantly higher compared to female respondents for all traits. For Machiavellianism, it was t(586) = −5.862, *p* < 0.001, d = −0.51 (M_males_ = 29.18, SD = 6.27 versus M_females_ = 26.10, SD = 5.98); for narcissism, it was t(586) = −3.498, *p* < 0.001, d = −0.30 (M_males_ = 24.49, SD = 4.98 versus M_females_ = 23.07, SD = 5.51); for psychopathy, it was t(586) = −9.443, *p* < 0.001, d = −0.82 (M_males_ = 21.98, SD = 5.91 versus M_females_ = 17.45, SD = 5.31).

We tested the factor structure of the Short Dark Triad using confirmatory factor analysis. For that purpose, the diagonally weighted least squares (DWLS) estimation method with robust correction was used, because it better examines the data that do not meet the criteria of normal distribution [42]. In order to verify different alternatives of model structure developed by authors of the original scale [1] or examined in studies of language modifications [15,31], three models were examined. Our priority was the three-factor model consisting of three equal intercorrelated factors, which is coherent with the original model [1]. Additionally, this model reached the most satisfactory results in several studies [15,16,31]. Next, according to Persson et al. [28] who tried to revisit the factor structure of the Short Dark Triad, we tested a bifactor model with three specific factors, and a unidimensional model with all 27 items loading on one factor.

The results of the confirmatory factor analysis are presented in Table 4. The chi-squared value was statistically significant for all models (*p* < 0.05). This result was not satisfactory for this type of analysis, but given the size of the research sample, we considered such a result as understandable. As can be seen in Table 4, the three-factor model achieved the best model fit. The ratio of the chi-squared value to degrees of freedom met the criteria, because the cut-off criterion was set at <3; some sources report an acceptable value of ≤5, which was met by the model. The comparative fit index (CFI) and the Tucker–Lewis index (TLI) achieved the required values, which were set at >0.9. The RMSEA index should fall in the range <0.05, but some sources state that the criterion <0.08 is also sufficient [43]. 

Item analysis found three problematic items. In the subscale of Machiavellianism, it was item M1 (“*It’s not wise to tell your secrets.*”), item N9 (“*I insist on getting the respect I deserve.*”) in the subscale of narcissism, and item P2 (“*I avoid dangerous situations.*”) in the subscale of psychopathy. Therefore, we conducted one more test of a three-factor model, without the problematic items. The resulting index values were only slightly better than with the original number of items (Table 4).

## 5. Study 2—Test–retest Reliability of the Slovak Short Dark Triad

The aim of the second study was to test the consistency of the results obtained from the administration of the scale over time. The scale should capture stable personality traits; therefore, there was a presumption that the results obtained from the administration of the scale by the same respondents over time should be significantly correlated. For this purpose, the test–retest reliability procedure was used.

### 5.1. Method

#### 5.1.1. Research Sample

The second study involved 117 participants aged 20 to 60 years (M_Age_ = 30.0; SD = 9.59). The gender representation of the respondents was as follows—men made up 28.2% (N = 33) of the research group and were aged 21 to 60 years (M_Age_ = 31.0; SD = 10.3); women made up 71.8% (N = 84) of the research population and were aged 20 to 55 years (M_Age_ = 28.9; SD = 8.88). A convenience and purposive sampling method was used. 

#### 5.1.2. Measures

In Study 2, the same 27-item version of the Short Dark Triad was used as in Study 1.

#### 5.1.3. Procedure and Statistical Analyses

Due to the aim of the study, a Short Dark Triad scale was administered to the respondents in February 2019 in two stages in the form of a pencil–paper assessment. Students and teachers from Slovak universities were contacted and they were asked not only to fill in the questionnaire but also to distribute it further. In the first stage, the respondents were first informed that the completion of the questionnaire was voluntary, and the data would be processed only in this research study. All respondents agreed to participate in the survey. Subsequently, they were asked to provide their unique code before starting the questionnaire. This code was used to match the completed questionnaires from both research phases. The code took the form of their mother’s maiden name initials, the day of the participant’s birth and their father’s initials. Questionnaires were administered to respondents three weeks apart, which is recommended for testing the time stability of this type of test [44]. The collected data were subjected to statistical analysis in IBM SPSS Statistics 21 software.

There were no missing data from the data file. Testing the normality of the data distribution using the Kolmogorov–Smirnov test showed that the data were not normally distributed (*p* < 0.05), except for narcissism, where *p* = 0.200. The skewness value did not exceed the criterion >±1; the value of kurtosis exceeded this criterion in the subscale of psychopathy in both the test and retest. The internal consistency of the Short Dark Triad subscales was determined using McDonald’s omega in both phases. We described the data using other descriptive indicators (arithmetic mean, median, standard deviation, minimum, maximum). We evaluated the test–retest reliability of the Short Dark Triad subscales using the Spearman correlation coefficient (Spearman ρ) and intraclass correlation.

### 5.2. Results

Table 5 presents the values of the basic descriptive indicators for all three subscales of the Short Dark Triad—Machiavellianism, narcissism and psychopathy—in the test and retest.

The reliability of individual subscales for both the test and retest were calculated using McDonald’s omega. The subscale of Machiavellianism had a value in the test ω_m_ = 0.750; in the retest ω_mr_ = 0.754. The narcissism subscale had a value in the test ω_n_ = 0.697; in the retest ω_nr_ = 0.713. The psychopathy subscale had a value in the test ω_p_ = 0.655; in the retest ω_pr_ = 0.734.

Test–retest reliability of the components of the Short Dark Triad was calculated as the correlation of the scores collected from the first and second stages. The values of the stability coefficient for the Machiavellianism subscale were ρ_m_ = 0.843 (*p* < 0.001; 95% CI [0.781; 0.888]); for the narcissism subscale they were ρ_n_ = 0.791 (*p* < 0.001; 95% CI [0.712; 0.850]); and for the psychopathy subscale they were ρ_p_ = 0.625 (*p* < 0.001; 95% CI [0.501; 0.724]). We also tested the data using intraclass correlation, where the coefficient for the Machiavellianism subscale was 0.853 (*p* < 0.001; 95% CI [0.794; 0.896]), for the narcissism subscale it was 0.798 (*p* < 0.001; 95% CI [0.721; 0.855]), and for psychopathy subscale it was 0.686 (*p* < 0.001; 95% CI [0.577; 0.771]). All values of the stability coefficients were significant and high, which indicates good reliability of the test in terms of time stability.

## 6. Study 3—Convergent and Divergent Validity of the Slovak Short Dark Triad

The aim of the third study was to test the validity of the Short Dark Triad using the NEO-FFI Five-Factor Personality Inventory [2]. In the literature, we found several references which indicated that the individual components of the Dark Triad are related to the components of the Big Five—some constructs are similar, some are contrasting [4,15,16,17,45].

### 6.1. Method

#### 6.1.1. Research Sample

The third study involved 333 participants aged 17 to 65 years (M_Age_ = 26.5 SD = 11.37). The gender representation of the respondents was as follows—men made up 45% (N = 150) of the research group and were aged 17 to 62 years (M_Age_ = 23.0; SD = 9.14); women made up 55% (N = 183) of the research population and were aged 17 to 65 years (M_Age_ = 30; SD = 13.6). A convenience and purposive sampling method was used. 

#### 6.1.2. Measures

In Study 3, the same 27-item version of the Short Dark Triad was used as in Study 1 and Study 2.

For the purpose of capturing individual personality differences, the NEO-FFI Five-Factor Personality Inventory [2] was used, which represents an adapted Slovak version of the NEO Five-Factor Personality Inventory [46]. The inventory consists of 60 statements, where the role of the respondent is to express the extent of their agreement for each statement on a 5-point Likert scale (0 = it does not apply to me at all, 4 = it applies to me completely). These items saturated five basic subscales reflecting personality factors according to the Big Five model, with each of the scales corresponding to 12 items. The inventory captured the dimension of neuroticism (e.g., “*I often feel worse than other people.*”), extraversion (e.g., “*I like to have a lot of people around me.*”), openness to experience (e.g., “*When I read a book or look at a work of art, I sometimes feel chills or enthusiasm.*”), conscientiousness (e.g., “*I can organize my time well so that I can handle all the necessary matters in time.*”) and agreeableness (e.g., “*I try to be friendly to everyone I meet.*”). The total score has been calculated separately for each of the dimensions, representing the sum of the points obtained in the individual items. The lowest number of points that could be achieved was 0, and the highest was 48, for each of the scales. Before calculating the total score, it was necessary to reverse-code 27 items.

#### 6.1.3. Procedure and Statistical Analyses

The questionnaires were administered to the respondents in the period March 2019–April 2019 in two different forms. The electronic version of the questionnaires was created using the Google Docs-Form web application and sent via e-mail. The second version was in printed form (“pencil-paper”). Students and teachers from Slovak universities were contacted and they were asked not only to fill in the questionnaire but also to distribute it further. Respondents were informed that the completion of the questionnaire was voluntary and anonymous, and the data would be processed only in this research study. All respondents agreed to participate in the research.

The collected data were subjected to statistical analysis in IBM SPSS Statistics 21. There were no missing data from the data file. Testing the normality of the data distribution using the Kolmogorov–Smirnov test showed that the data were not normally distributed (*p* < 0.05). The value of skewness and kurtosis did not exceed the criterion >±1. We described the obtained data using other descriptive indicators (arithmetic mean, median, standard deviation, minimum, maximum). Convergent and divergent validity were determined using the Spearman correlation coefficient (Spearman ρ); partial correlation analysis was conducted as well.

### 6.2. Results

Table 6 shows the values of the basic descriptive indicators for all three subscales of the Short Dark Triad—Machiavellianism, narcissism, and psychopathy—and five personality factors—neuroticism, extraversion, openness, conscientiousness, and agreeableness. 

Convergent validity showed a weak, but significant, negative relationship of Machiavellianism with openness (*p* < 0.001; 95% CI [0.091; 0.297]) and conscientiousness (*p* < 0.001; 95% CI [0.077; 0.283]), and a moderately significant negative relationship with agreeableness (*p* < 0.001; 95% CI [0.440; 0.596]). We found that narcissism and psychopathy had an effect on the relationship of Machiavellianism with extraversion and conscientiousness. Partial correlation resulted in a weak but significant negative relationship with openness (*p* = 0.017), agreeableness (*p* < 0.001) and extraversion (*p* = 0.016). 

Additionally, a weak but significant negative relationship was identified between narcissism and neuroticism (*p* < 0.001; 95% CI [0.091; 0.297]) and agreeableness (*p* < 0.001; 95% CI [0.155; 0.355]), and a moderate significant positive relationship with extraversion (*p* < 0.001; 95% CI [0.217; 0.410]). In addition, Machiavellianism and psychopathy affected the relationships of narcissism with agreeableness and conscientiousness. Partial correlation resulted in a moderate negative significant relationship with neuroticism (*p* < 0.001) and positive moderate significant relationships with extraversion (*p* < 0.001) and conscientiousness (*p* = 0.002).

Psychopathy was slightly negatively correlated with openness (*p* < 0.001; 95% CI [0.0807; 0.286]) and conscientiousness (*p* < 0.001; 95% CI [0.205; 0.399]), and had a strong significant negative relationship with agreeableness (*p* < 0.001; 95% CI [0.552; 0.683]). We also found that narcissism and Machiavellianism had an effect on the relationship of psychopathy with neuroticism and extraversion. Partial correlation showed a moderate positive significant relationship with neuroticism (*p* = 0.002) and moderate negative significant relationships with extraversion (*p* = 0.015), openness (*p* = 0.036), agreeableness (*p* < 0.001) and conscientiousness (*p* < 0.001).

In the case of divergent validity, no significant or strong relationship of Machiavellianism with extraversion and neuroticism was demonstrated. After controlling the effect of narcissism and psychopathy, no relationships with neuroticism and conscientiousness were found. Narcissism did not have a significant or strong relationship with openness or conscientiousness. Partial correlation showed no relationship with openness and agreeableness. Psychopathy had no relationship with neuroticism or extraversion. However, after controlling the effect of Machiavellianism and narcissism, all correlations became significant. We evaluate these results as satisfactory; thus, the goal of the third study was fulfilled. The exact values of the correlation coefficients with the significant values highlighted are presented in Table 7.

## 7. Discussion 

The aim of the present study was to verify the psychometric properties of the Slovak Short Dark Triad scale, which originated as a Slovak version of the original Short Dark Triad [1]. This verification was performed in three studies. The first two studies aimed to examine the reliability of the scale—internal consistency, consistency over the time, and factor structure. The third study aimed to verify convergent and divergent validity. We consider the objectives of all three studies to be fulfilled. Study 1 focused on verifying internal consistency, factor structure, and intercorrelations between subscales. The coefficients of internal consistency yielded satisfactory results comparable to the results of the original English scale [1], as well as its other language modifications [15,16]. Some items in the subscales did not correlate ideally with the total score of individual subscales; therefore, it would be appropriate to consider whether it would be suitable to exclude three items (one from each subscale) from the analyses and work only with the 24-item version of the scale. Such a procedure could be found with both the Czech version [16] and the Argentine version [31]. In contrast, in the German version [15] and the Turkish version [34], the authors maintained the original 27-item version despite the similar final values. McDonald’s omega values would only increase slightly after excluding items; therefore, we decided to use a full set of items for further analysis.

The values of correlation coefficients in testing the intercorrelations between the subscales showed values that reflected the theoretical conceptualization of the Dark Triad as a phenomenon of overlapping aversive personality traits. The subscales correlated significantly with each other, the relationship between Machiavellianism and psychopathy appeared to be the strongest, and the relationships between Machiavellianism and narcissism, and between narcissism and psychopathy, were moderately strong. The nature of these relationships corresponds to the values of correlation coefficients in the original scale [1], as well as its Czech [16] and German versions [15].

The results of gender difference testing correlated with the results of previous studies, where male respondents scored more highly in all three dark traits compared to female respondents [1,14,15,42,47].

The results of confirmatory factor analysis supported factor saturation of subscales according to the original three-factor model [1]. Using polychoric correlations with the DWLS method, acceptable values of model fit were achieved. Excluding three problematic items resulted only in negligible improvement. A significant chi-squared value occurred in our study, similarly with the verification of other language modifications [15,16,34]. There are several reasons why some items have proven to be problematic. Naturally, this could be due to the translation of the original questionnaire into Slovakian. Another possible problem could have been negatively formulated statements, which in the Slovak language could complicate the respondents’ understanding of the meaning of the item. From the theoretical basis and our correlation analysis, it was also clear that the Dark Triad phenomenon traits overlapped, sharing certain characteristics. Therefore, it is possible that items which reduced the reliability of subscales partially saturated some other aversive features.

Study 2 aimed to verify the consistency of the results over time. The values of correlation coefficients were significant and high in all three cases, as in the Czech [16], German [15] and Turkish [34] versions of the scale.

Study 3 aimed to verify the validity. As stated by Jones and Paulhus [1], the basic step in verifying the validity of the results is to find out how the methodology works in comparison with the so-called gold standard—proven and used methodologies. For this reason, the obvious choice was the Five-Factor Personality Inventory [2]. Verification of the nature of the relationships between the components of the Dark Triad and the components of the Big Five model has been utilized since the conceptualization of the Dark Triad itself [4], and is part of other studies [13,15,16,17,45]. More comprehensive information is provided in the review by Furnham et al. [14]. In the case of convergent validity, we state that the results corresponded to the theoretical basis [4] and to the findings of other authors. In particular, a significant relationship of Machiavellianism with agreeableness [4,13,15,16,17] and conscientiousness has been confirmed [4,13,15] as well as significant relationships of narcissism with neuroticism [16,17], extraversion [4,13,16,17] and agreeableness [4,13,15,16], and significant relationships of psychopathy with openness [4], agreeableness [4,13,15,16,17] and conscientiousness [4,13,15,16] have also been confirmed. The divergent validity correlated with the findings of Paulhus and Williams [4], who also did not predict strong or significant relationships of Machiavellianism with neuroticism and extraversion, or narcissism with conscientiousness. In our study, narcissism did not correlate with openness, and psychopathy did not correlate with neuroticism and extraversion. The components of the Dark Triad had negative relationships with the components of the Big Five in almost all analyses, which is not surprising if we realize that, for example, Machiavellianism as a trait showing manipulative tactics or an effort to exploit people is in stark contrast to agreeableness (a positive attitude towards people or conscientiousness based on ethical and moral principles). Narcissism had the strongest positive relationship with extraversion, which is the result of a narcissistic effort to demonstrate its own perfection and desire to draw attention to itself. Psychopathy showed the strongest negative relationship with agreeableness, which accurately reflects the coldness in the interpersonal relationships of individuals with psychopathic personality structure. Finally, it should be noted that the Short Dark Triad was correlated with NEO-FFI in the German Short Dark Triad scale [15] and in the research of the Dark Triad on an adolescent sample [45]. In other studies, the Dirty Dozen methodology [13] or methodologies that capture Machiavellianism, narcissism, and psychopathy separately [4] have been used instead of the Short Dark Triad. Instead of NEO-FFI, modifications such as BF-44 [16] or Mini-International Item Pool [17] were used.

The result of the analyses performed in all three studies was a 27-item Short Dark Triad Scale (Appendix A) consisting of three nine-item subscales—Machiavellianism, narcissism and psychopathy. The statements were rated on a 5-point Likert scale (1 = strongly disagree; 5 = I strongly agree). The final score was calculated as the sum of points obtained for each subscale separately, although before the sum was calculated, it was necessary to reverse-code five items.

A possible limitation of our study could be the not-so-robust confirmatory factor analysis results. In the future, we certainly recommend subjecting the Slovak Short Dark Triad scale to further testing and to consider whether it would be more appropriate to shorten it by excluding items that showed problematic values (however, it should be noted that the values were not critical enough to significantly affect the reliability or validity of the scale). The self-explanatory nature of the scale and the unmistakable formulations of individual items are also limiting, making it relatively easy for respondents to reveal that the items were related to their negative aspects, which people have a problem acknowledging due to natural efforts to maintain a positive self-image [48]. However, this is not only a problem of the Slovak version; its wording follows from the wording of the items in the original version [1]. A certain distortion of the results could also have been caused by the two techniques of distributing the questionnaires, because both electronic and classic administration have their advantages and disadvantages. Similarly to other studies [15,16,36,38,42], our study faced gender disproportion, although it is questionable to what extent the results may have been influenced by the unequal gender distribution, because men appeared to score more highly on the features of the Dark Triad than women [1,47]. Due to the high number of respondents in the research sample, the age range of respondents was large, but the median values of age in individual studies ranged from 23 to 25 years, which corresponded to the age of young adulthood [49]. As noted by Bratek et al. [50], it is necessary to consider the dynamics of the intensity of manifestations of aversive personality traits, which decrease with age.

Despite the obvious limitations, we consider the objectives of all three studies to have been met. In three studies, we verified the reliability (internal consistency, consistency over time, and factor structure) and validity (convergent and divergent) of the Slovak version of the Short Dark Triad in several ways. We consider the Slovak adaptation of the Short Dark Triad as a useful tool for Slovak settings. The results of our study are comparable with the results of other studies that verified the psychometric properties of the Short Dark Triad in other language variations. The original scale is intended for the non-clinical adult population; therefore, following the example of other researchers, we propose to focus on specific samples of respondents, such as adolescents [45,51], youths [52] or at-risk youths [53] in the future.

## 8. Conclusions

The Short Dark Triad [1] is popular tool used worldwide for capturing three aversive traits—Machiavellianism, narcissism and psychopathy. Its popularity has been proven by the existence of several language modifications across the world. However, until now, the Slovak translation was absent. 

We see the benefit of the Slovak version in the extension of the database of language modifications for the Short Dark Triad study. The Slovak version has the potential to be-come a counterpoint to the Five-Factor Personality Inventory, which has been adapted in Slovak conditions since 2007 [2]. From the available information, it is clear that the concept of the Dark Triad is interesting for the scientific community, and therefore it is appropriate to find and create a reliable and valid methodology by which this phenomenon could be captured. In our opinion, the Short Dark Triad also has good potential in Slovak settings due to the relatively small number of items and simple interpretation. We see the potential for further research in the already mentioned possible modifications of the scale and subsequent verification of its functionality in the Slovak population.

## Figures and Tables

**Table 1:** Comparison of Dark Triad and Big Five traits.

	MACHIAVELLIANISM		NARCISSISM		PSYCHOPATHY	
	+	−	+	−	+	−
Jakobwitz and Egan (2006)	N	C, A	E, O, C	A	N	C, A
Furnham et al. (2013)		C, A		C, A		C, A
Paulhus and Williams (2002)		C, A	E, O	A	E, O,	N, C, A
Malesza et al. (2019)		C, A	C	A		C, A
Odiakosa (2018)		A	E	N	N	A
Egorova and Adamovich (2019)		A	E, O	N	N, E	A

E, extraversion; O, openness; A, agreeableness; C, conscientiousness; N, neuroticism

**Table 2:** Descriptive characteristics of the Short Dark Triad subscales.

	MACH	NAR	PSY
N_R_	588	588	588
M	27.10	23.60	19.00
SD	6.25	5.38	5.92
Me	27.00	24.00	18.00
Min	11.00	9.00	9.00
Max	43.00	42.00	39.00
Skew	0.02	0.16	0.69
Kurt	−0.51	−0.01	0.15

MACH, Machiavellianism; NAR, narcissism; PSY, psychopathy; NR, number of respondents; M, mean; SD, standard deviation; Me, median; Min, minimum; Max, maximum; Skew, skewness; Kurt, kurtosis.

**Table 3:** Item analysis.

Item	Factor	M	SD	β	r	*ω*
M1	MACH 0.733	3.88	1.01	0.127	0.101	0.751
M2		2.37	1.26	0.573	0.446	0.702
M3		2.82	1.23	0.652	0.576	0.679
M4		3.27	1.28	0.346	0.320	0.724
M5		2.94	1.36	0.730	0.615	0.668
M6		1.95	1.24	0.642	0.456	0.701
M7		3.22	1.20	0.458	0.468	0.703
M8		3.19	1.38	0.436	0.387	0.714
M9		3.51	1.12	0.274	0.230	0.738
N1	NAR 0.667	2.68	1.13	0.509	0.444	0.631
N2		2.74	1.21	0.318	0.308	0.659
N3		2.48	1.12	0.582	0.409	0.635
N4		2.13	1.09	0.556	0.413	0.635
N5		3.31	1.18	0.456	0.384	0.646
N6		2.62	1.15	0.229	0.216	0.677
N7		1.77	1.01	0.568	0.407	0.636
N8		2.52	1.22	0.361	0.351	0.652
N9		3.37	1.18	0.273	0.163	0.685
P1	PSY 0.728	1.79	1.07	0.609	0.480	0.687
P2		2.65	1.25	0.131	0.112	0.745
P3		1.75	1.08	0.627	0.474	0.685
P4		2.15	1.23	0.434	0.420	0.703
P5		3.14	1.33	0.465	0.419	0.701
P6		2.29	1.23	0.641	0.493	0.683
P7		1.87	1.43	0.188	0.177	0.737
P8		1.47	1.05	0.480	0.422	0.702
P9		1.90	1.14	0.623	0.472	0.689

M1–M9, items in the Machiavellianism subscale; N1–N9, items in the narcissism subscale; P1–P9, items in the psychopathy subscale; MACH, Machiavellianism; NAR, narcissism; PSY, psychopathy; M, mean; SD, standard deviation; β, item factor loading; r, discriminating power of the item; ω, McDonald’s omega.

**Table 4:** Confirmatory factor analysis of the Slovak Short Dark Triad.

Model	N	χ^2^	df	*p*	χ^2^/df	CFI	TLI	RMSEA	RMSEA 90% CI	
									LB	UB
3-factor	588	670.023	321	<0.0001	2.08	0.95	0.94	0.062	0.055	0.068
1-factor	588	1207	324	<0.0001	3.72	0.72	0.69	0.068	0.064	0.072
bifactor	588	779.133	297	<0.0001	2.69	0.84	0.82	0.053	0.048	0.057
*3-factor-R*	*588*	*474.721*	*248*	*<0.0001*	*1.91*	*0.96*	*0.96*	*0.057*	*0.049*	*0.064*

N, number of respondents; χ^2^, chi square; df, degrees of freedom; χ^2^/df, ratio of chi-square value to degrees of freedom; CFI, comparative fit index; TLI, Tucker–Lewis index; RMSEA, root-mean-square error of approximation; CI, confidence interval; LB, lower bound; UB, upper bound.

**Table 5:** Descriptive characteristics of Short Dark Triad subscales in the test and retest.

	MACH	NAR	PSY	MACH_R_	NAR_R_	PSY_R_
N_R_	117	117	117	117	117	117
M	27.40	23.00	17.90	26.80	23.00	17.70
SD	6.35	5.34	4.92	5.83	5.30	5.26
Me	28.00	23.00	17.00	26.00	24.00	17.00
Min	14.00	11.00	9.00	15.00	11.00	9.00
Max	41.00	36.00	37.00	44.00	37.00	37.00
Skew	0.05	0.19	0.90	0.21	−0.13	0.73
Kurt	−0.59	−0.32	1.56	−0.30	−0.11	1.08

MACH, Machiavellianism; NAR, narcissism; PSY, psychopathy; MACH_R_, Machiavellianism retest; NAR_R_, narcissism retest; PSY_R_, psychopathy retest; N_R_, number of respondents; M, mean; SD, standard deviation; Me, median; Min, minimum; Max, maximum; Skew, skewness; Kurt, kurtosis.

**Table 6:** Descriptive characteristics of the Short Dark Triad subscales and the Big Five subscales.

	MACH	NAR	PSY	N	E	O	A	C
N_R_	333	333	333	333	333	333	333	333
M	27.10	23.80	19.40	35.60	40.60	39.00	42.60	44.70
SD	6.26	5.41	6.15	8.82	6.95	7.15	6.45	8.36
Me	27.00	24.00	19.00	36.00	41.00	39.00	43.00	45.00
Min	11.00	9.00	9.00	12.00	15.00	18.00	21.00	21.00
Max	43.00	42.00	39.00	60.00	57.00	57.00	57.00	60.00
Skew	0.04	0.12	0.60	0.12	−0.27	0.05	−0.25	−0.29
Kurt	−0.50	0.07	−0.10	−0.20	−0.01	−0.38	−0.15	−0.45

MACH, Machiavellianism; NAR, narcissism; PSY, psychopathy; N, neuroticism; E, extraversion; O, openness; A, agreeableness; C, conscientiousness; NR, number of respondents; M, mean; SD, standard deviation; Me, median; Min, minimum; Max, maximum; Skew, skewness; Kurt, kurtosis.

**Table 7:** Convergent and divergent validity of the Slovak Short Dark Triad.

	N	E	O	A	C
MACH	0.065	−0.069	−0.196 **	−0.522 **	−0.182 **
*partial*	0.037	−0.116 *	−0.115 *	−0.183 ***	−0.039
NAR	−0.196 **	0.317 **	−0.065	−0.258 **	0.034
*partial*	−0.258 ***	0.401 ***	0.057	0.054	0.146 **
PSY	0.076	−0.017	−0.185 **	−0.622 **	−0.305 **
*partial*	0.149 *	−0.117 *	−0.101 *	−0.482 **	−0.242 ***

MACH, Machiavellianism; NAR, narcissism; PSY, psychopathy; N, neuroticism; E, extraversion; O, openness; A, agreeableness; C, conscientiousness; * *p* < 0.01 (Sig. 2-tailed); ** *p* < 0.01 (Sig. 2-tailed); *** *p* < 0.01 (Sig. 2-tailed).

## Data Availability

The data are not publicly available.

## Appendix A

### Appendix A.1. KRÁTKA ŠKÁLA TEMNEJ TRIÁDY (Short Dark Triad)

Na predloženej škále (1 = vôbec nesúhlasím; 5 = úplne súhlasím) zaznačte, do akej miery súhlasíte s jednotlivými výrokmi. *(Please indicate how much you agree with each of the following statements; 1 = disagree strongly; 5 = agree strongly.)*

Poznámka: zvýraznené položky je nutné prepólovať; škálu používať bez označenia subškál. *(reverse-coded items in bold)*

### Appendix A.2. MACHIAVELIZMUS(Machiavellianism)

1. Nie je múdre prezrádzať svoje tajomstvá.
  *(It’s not wise to tell your secrets.)*
2. Rád šikovne manipulujem ľuďmi, aby som dosiahol, čo chcem.
  *(I like to use clever manipulation to get my way.)*
3. Nech to stojí čokoľvek, dôležitých ľudí musíš dostať na svoju stranu.
  *(Whatever it takes, you must get the important people on your side.)*
4. Je dobré vyhýbať sa priamemu konfliktu s inými ľuďmi, pretože v budúcnosti môžu byť užitoční.
  *(Avoid direct conflict with others because they may be useful in the future.)*
5. Je múdre pamätať si nejaké informácie, ktoré môžeme neskôr použiť proti ostatným.
  *(It’s wise to keep track of information that you can use against people later.)*
6. Mali by sme počkať na správny čas, kedy ľuďom vrátime to zlé, čo urobili oni nám.
  *(You should wait for the right time to get back at people.)*
7. Sú veci, ktoré by si mal pred ostatnými skrývať, aby si si udržal svoju povesť.
  *(There are things you should hide from other people to preserve your reputation.)*
8. Uisti sa, že tvoje plány sú prospešné pre teba a nie pre druhých.
  *(Make sure your plans benefit yourself, not others.)*
9. Väčšina ľudí sa dá zmanipulovať.
  *(Most people can be manipulated.)*

### Appendix A.3. NARCIZMUS (narcissism)

10. Ľudia ma vnímajú ako prirodzeného lídra/vodcu.
  *(People see me as a natural leader.)*
11. **Neznášam byť stredobodom pozornosti. **
  ***(I hate being the center of attention.)***
12. Mnohé skupinové aktivity zvyknú byť bezo mňa nudné.
  *(Many group activities tend to be dull without me.)*
13. Viem, že som výnimočný/á, pretože mi to každý neustále hovorí.
  *(I know that I am special because everyone keeps telling me so.)*
14. Rád sa zoznamujem s dôležitými ľuďmi.
  *(I like to get acquainted with important people.)*
15. **Cítim sa rozpačito, keď mi niekto lichotí. **
  ***(I feel embarrassed if someone compliments me.)***
16. Prirovnávajú ma k slávnym ľuďom.
  *(I have been compared to famous people.)*
17. **Som priemerný človek. **
  ***(I am an average person.)***
18. Trvám na tom, aby som získal rešpekt, aký si zaslúžim.
  *(I insist on getting the respect I deserve.)*

### Appendix A.4. PSYCHOPATIA (psychopathy)

19. Rád/a sa mstím autoritám.
  *(I like to get revenge on authorities.)*
20. **Vyhýbam sa nebezpečným situáciám.**
  ***(I avoid dangerous situations.)***
21. Odplata musí byť rýchla a nepríjemná.
  *(Payback needs to be quick and nasty.)*
22. Ľudia často hovoria, že sa neviem kontrolovať.
  *(People often say I’m out of control.)*
23. Je pravda, že viem byť k iným zlý.
  *(It’s true that I can be mean to others.)*
24. Ľudia, ktorí sa so mnou zahrávajú, to potom vždy oľutujú.
  *(People who mess with me always regret it.)*
25. **Nikdy som sa nedostal/a do problémov so zákonom.**
  ***(I have never gotten into trouble with the law.)***
26. Užívam si sex s ľuďmi, ktorých sotva poznám.
  *(I enjoy having sex with people I hardly know.)*
27. Poviem čokoľvek, len aby som dosiahol to, čo chcem.
  *(I’ll say anything to get what I want.)*
